# Association of peripheral arterial disease with all-cause and cardiovascular mortality in hemodialysis patients: a meta-analysis

**DOI:** 10.1186/s12882-016-0397-1

**Published:** 2016-11-25

**Authors:** Yi Yang, Yong Ning, Weifeng Shang, Ran Luo, Lixi Li, Shuiming Guo, Gang Xu, Xiaofeng He, Shuwang Ge

**Affiliations:** 1 Department of Nephrology, Tongji Hospital Affiliated with Tongji Medical College, Huazhong University of Science and Technology, Wuhan, Hubei 430030 People’s Republic of China; 2 Department of Nephrology, Puai Hospital, Tongji Medical College, Huazhong University of Science and Technology, Wuhan, Hubei 430000 People’s Republic of China

**Keywords:** Peripheral arterial disease, Hemodialysis, Mortality, Meta-analysis

## Abstract

**Background:**

Recent studies have shown an association between peripheral arterial disease (PAD) and increased risk of mortality in hemodialysis (HD) patients; however, the estimates vary widely and are inconsistent. It is necessary to elucidate the degree of mortality risk for PAD patients in HD population.

**Methods:**

PubMed, EMBASE, Web of Science and Cochrane Library (from inception to September 4th, 2016) were systematically searched for cohort studies assessing the association between PAD and mortality in HD patients. We calculated the pooled risk ratios (RRs) with 95% confidence intervals (CI) of all-cause and cardiovascular (CV) mortality using random effects models. Subgroup analyses were conducted to explore the source of heterogeneity.

**Results:**

The search identified 2,973 potentially eligible records and 10 studies (*n* = 32,864) were included. Our meta-analysis revealed that PAD significantly increased the risk of all-cause mortality (RR 2.15, 95 % CI 1.67–2.77, *n* = 32,864) and CV mortality (RR 2.99, 95 % CI 1.66-5.38, *n* = 31,794) in HD patients after multivariate adjustment. Subgroup analyses showed the study design and follow-up time might be two sources of heterogeneity.

**Conclusion:**

PAD may be a prognostic marker of all-cause and CV mortality in HD patients. More attention should be paid to diagnosis and management of PAD in HD patients.

**Electronic supplementary material:**

The online version of this article (doi:10.1186/s12882-016-0397-1) contains supplementary material, which is available to authorized users.

## Background

More than 2 million people are suffering from end-stage renal disease (ESRD) worldwide [1] and growing numbers of patients are receiving hemodialysis (HD) as a choice for renal replacement therapy [2]. The mortality of HD patients was high. A review reported that the annual crude death rate was in the range of 9.0%–10.2% for dialysis populations in Japan [2]. Yan et al. [3] followed up 385,074 HD patients and reported that the one-year mortality was 19.8% and the five-year mortality was 43.0%. The high mortality of HD may be due to the plenty of vascular complications, such as vascular calcification [4] and atherosclerosis [5]. Peripheral arterial disease (PAD) is a systemic vascular disorder involving the aorta, iliac, and lower extremity arteries usually secondary to atherosclerosis [6, 7]. It is diagnosed by ankle-brachial/arm blood pressure index (ABI, or AAI, or ABPI) measurement and clinical assessment. ABI is the ratio of ankle to brachial systolic blood pressure and patients with an ABI value < 0.9 can be diagnosed as PAD. The main clinical manifestations of PAD contains intermittent claudication and critical limb ischemia.

An association between PAD and mortality in HD patients has been reported since 1916 and the multivariable-adjusted RRs (95% CI) were 2.9 (1.1–6.7) for all-cause and 7.1 (1.8–33.3) for cardiovascular (CV) mortality [8]. Recent several studies [9–17] had the consistent outcomes with the article in 1996 for all-cause mortality. But for CV mortality, the outcomes were inconsistent. Tsai et al. observed 444 participants with a mean follow-up of 51.5 months and found that PAD was not significantly associated with CV mortality in HD patients [12]. Meanwhile, the estimates of the associated risk varied widely.

Given the varied and inconsistent results, a meta-analysis was conducted to summarize the association between PAD and the risk of all-cause and CV mortality in patients undergoing HD.

## Methods

Our study was designed, conducted, and reported based on Preferred Reporting Items for Systematic Reviews and Meta-Analyses (PRISMA) guidelines (Additional file 1) [18]. Databases (PubMed, EMBASE, Web of Science, Cochrane Library) were systematically screened to identify relevant records published up to September 4th, 2016 (Additional file 2) using the following term (Peripheral artery disease OR Peripheral arterial disease OR PAD OR Claudication OR limb ischaemia OR limb ischemia OR ABI OR ABPI OR ankle-brachial index OR Ankle-brachial blood pressure index OR peripheral arterial occlusive disease) AND (peritoneal dialysis OR hemodialysis OR dialysis) AND (Mortality OR Death OR Outcome OR Prognos*). Besides, we reviewed references of all articles identified and previous reviews on this topic for additional further relevant studies. Two authors (Y.Y. and Y.N.) separately screened titles and abstracts, checked full-text articles, and determined the final eligible records. If the eligibility of a record was uncertain, the full text was reviewed. We resolved divergences by discussion or remitting to a third reviewer, and asked the authors for help when necessary.

### Inclusion criteria

Studies included should meet all of the following criteria: (1) cohort study, (2) general HD population (i.e., no other specific diseases, not be given special operation or interventional therapy), (3) at start, patients with PAD were recognized using definition of criteria for PAD, and/or ABI was measured at baseline using a technique standardized in each study. We considered the patients with ABI < 0.9 as having PAD, (4) studies had analysis for association between patients with PAD and the risk of all-cause and/or CV mortality on follow-up, and quantitative data evaluating the association was possible to be extracted, (5) there was no language restriction.

### Exclusion criteria

Studies should be excluded by the following criteria: case–control or cross-sectional studies,animal studies, reviews, editorials, meeting abstracts, correction, and meta-analyses. In cases where several publications used the same or overlapping datasets, we used the study with most thorough information about mortality risk associated with patients with PAD versus controls.

### Data extraction

The following information were extracted by two authors separately using a uniform prepared data extraction form: name of first author, publication year, country of origin, study design, sample size, mean age, proportion of men, follow-up time, duration of dialysis, prevalence of diabetes mellitus, prevalence of PAD, diagnosis criterion of PAD, and events for analysis. We contacted to the original author in the case of having any doubt. Additional information was included if response was obtained, otherwise, we used the available data for our analyses.

### Quality assessment

The internal validity (potential bias and methodological quality) of each study included was appraised by 2 authors independently (Y.Y. and Y.N.) according to the Newcastle-Ottawa Scale (NOS) [19] for cohort studies which assessed the following: (1) exposed cohort truly or somewhat representative, (2) non-exposed cohort drawn from the same community as the exposed cohort, (3) ascertainment of exposure, (4) outcome of interest not present at start, (5A) study controls for age; (5B) study controls for ≥ 3 additional risk factors, (6) assessment of outcome (independent blind assessment or record linkage), (7) follow-up ≥ 62 m, (8) complete accounting for cohorts or subjects lost to follow-up unlikely to introduce bias. This scale assesses the quality of observational studies and allocates a maximum of 9 points for quality of study participants. Overall study quality was graded as good (score, 7–9), fair (score, 4–6), or poor (score, 0–3) [20]. Divergence was resolved through discussion or consensus.

### Statistical analyses

The studies included in our meta-analysis reported different effect measure (hazard ratio [HR] or risk ratio [RR]), and we combined it as RR throughout this article. Multivariable-adjusted all-cause and CV mortality data were expressed as RRs and corresponding 95% CIs. Random effects models [21] were used to analyze pooled results of studies in view of high heterogeneity among our studies. Heterogeneity of RR across included studies was assessed with Chi-squared based on Q-statistic test (*P* < 0.1) and quantified using I^2^ index. Roughly, Higgins I^2^ values were interpreted as low (25%), moderate (50%), and high (75%) heterogeneity. To explore the source of heterogeneity, subgroup analysis and univariable random effects meta-regression were conducted. Publication bias was assessed using funnel plots by the Egger regression asymmetry test. Sensitivity analyses were conducted by recalculating the pooled RR with removal of one study once. All analyses were conducted with Stata 10.0 (College Station, TX, USA). A *P* value of < 0.05 by 2 tailed was set to be significant.

## Results

### Study selection and study characteristics

**Fig. 1:**
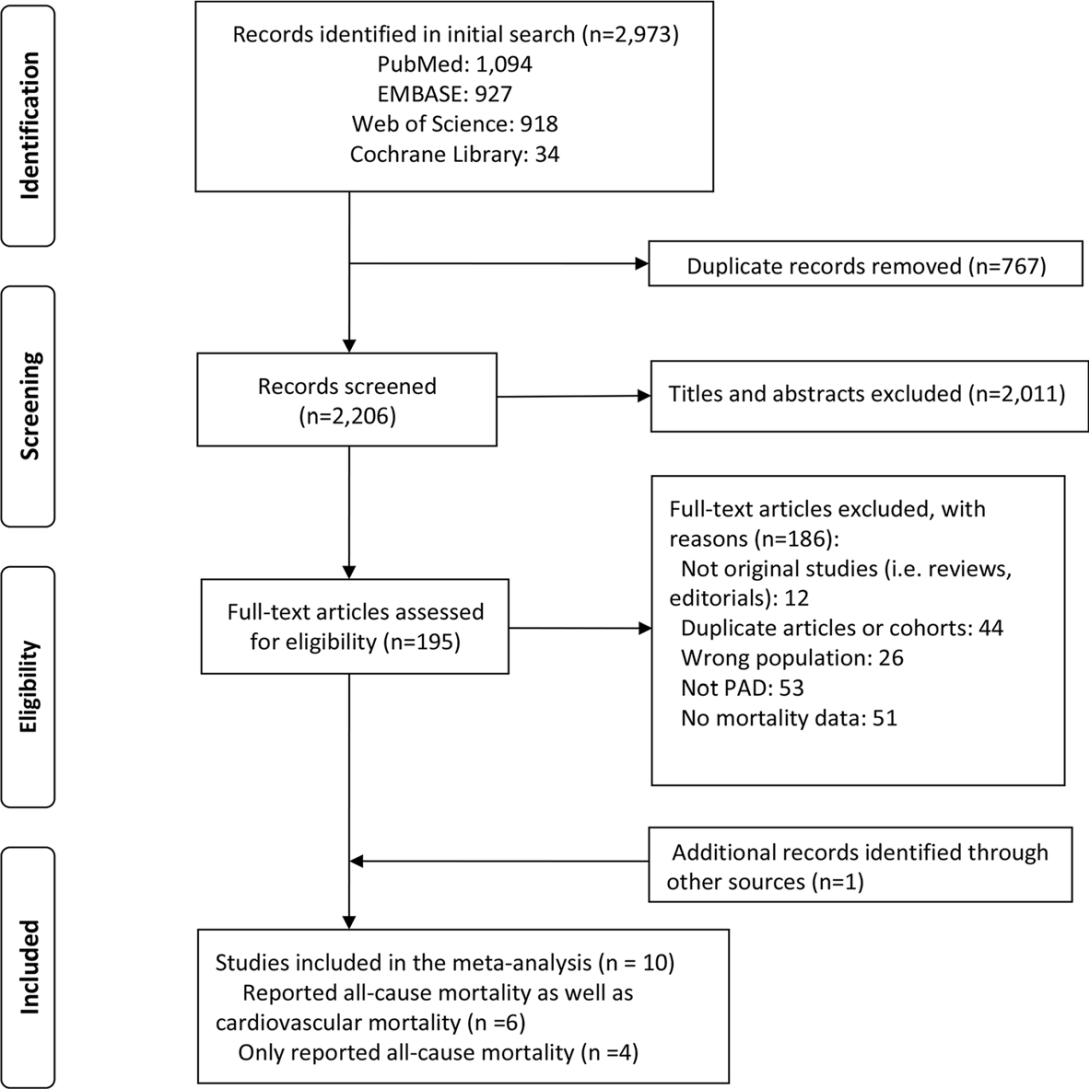
Flow diagram of selection of studies

We yielded 2,973 potentially relevant records initially through database searches, and 195 records were remained eventually for full-text review, of which 186 were excluded. And 1 additional study was retrieved using hand searching the reference lists of included studies. Therefore, 10 cohort studies were included in this meta-analysis [8–17], of which 10 studies were included for meta-analysis of all-cause mortality [8–17], and 6 were included for CV mortality [8–10, 12, 15, 16]. Figure 1 showed the summary of study identification process.

Table 1 showed the characteristics of the 10 included studies (*n* = 32,864). They were published from 1996 to 2015. 5 articles were from Asia [9, 13–16], 2 from the United States [8, 11], 2 from Europe [12, 17], and 1 multinational study from Asia, USA and Europe [10]. An ABI value < 0.9 was the single diagnostic criteria of PAD in 4 studies [8, 9, 13, 15]. Of the 10 articles included, 8 reported the means duration of dialysis (min: 5.8 years; max: 19.8 years) [9, 11–13, 15–17]. For the 6 studies (2 were retrospective, and 4 were prospective, *n* = 31,794) included to analyze CVD mortality [8–10, 12, 15, 16], only 1 study did not use ABI < 0.9 to diagnose PAD [10].

**Table 1:** Characteristics of included studies

Study	Country	Design	Sample size	Mean age (years)	Men (%)	Follow up time (months)	Duration of dialysis (years)	Diabetes mellitus (%)	PAD (%)	Diagnosis of PAD	Events for analysis
Fishbane et al. 1996 [8]	USA	PC	132	61.5	59	12	NA	25.0	35.0	ABI	AC/CV mortality
Ono et al. 2003 [9]	Japan	PC	1010	60.6	63.5	24	6.5	33.8	16.5	ABI	AC/CV mortality
Rajagopalan et al. 2006 [10] Vega et al. 2008 [17]	Multi-nation Spain	RC RC	29873 220	61.4 62	57.7 56	64.8 47	NA 5.8	37.7 21.4	25.3 40.5	Clinical ssessment, history Clinical assessment, doooler	AC/CV mortality AC mortality
Cohen et al. 2010 [11]	USA	PC	512	61	55.9	6	19.8	NA	3.3	NA	AC mortality
Adragao et al. 2012 [12]	Portugal	PC	219	65	60	36	6.8	20.0	41.0	ABI, vascular calcification	AC/CV mortality
Otsubo et al. 2012 [13]	Japan	RC	86	59.8	69.8	105.6	15.1	19.8	22.1	ABI	AC mortality
Thani et al. 2013 [14]	Qatar	PC	252	57	50.3	36	7.3	59.2	38.5	ABI, Clinical assessment	AC mortality
Tsai et al. 2015 [15]	Taiwan, China	RC	444	61.6	46.4	79.2	7.7	32.7	24.8	ABI	AC/CV mortality
Zhou et al. 2015 [16]	China	PC	116	56.4	53.4	72	7.1	9.5	18.0	ABI, clinical assessment, history	AC/CV mortality

Abbreviations: *PAD* peripheral arterial disease; *USA* United states of America; *PC* prospective; *RC* retrospective; *NA*: not applicable; *ABI* ankle-brachial blood pressure index; *AC*: all-cause; *CV* cardiovascular. Multination^a^: United States,Europe,Japan, Canada, Australia/New Zealand

According to the NOS, the methodological quality of 9 studies included was graded as good and 1 study was graded as fair. The details were presented in Table 2.

**Table 2:** Assessment of study quality

References	Quality indications form of Newcastle-Ottawa Scale									Total stars
	1	2	3	4	5A	5B	6	7	8	
Fishbane et al. 1996 [8]	☆	☆	☆	☆	☆	☆	☆	-	☆	8
Ono et al. 2003 [9]	☆	☆	☆	☆	☆	☆	☆	-	☆	8
Rajagopalan et al.2006 [10] Vega et al.2008 [17]	☆ ☆	☆ ☆	☆ ☆	☆ ☆	☆ ☆	☆ -	☆ ☆	☆ -	☆ ☆	9 7
Cohen et al.2010 [11]	☆	☆	-	☆	☆	☆	☆	-	☆	7
Adragao et al. 2012 [12]	☆	☆	☆	☆	☆	☆	-	-	☆	7
Otsubo et al. 2012 [13]	☆	☆	☆	☆	☆	☆	☆	☆	-	8
Thani et al. 2013 [14]	☆	☆	☆	☆	☆	☆	-	-	-	6
Tsai et al. 2015 [15]	☆	☆	☆	☆	☆	☆	☆	☆	☆	9
Zhou et al. 2015 [16]	☆	☆	☆	☆	☆	☆	☆	☆	☆	9

For cohort studies: 1, exposed cohort truly or somewhat representative; 2, nonexposed cohort drawn from the same community as the exposed cohort; 3, ascertainment of exposure; 4, outcome of interest not present at start; 5A, study controls for age; 5B, study controls for ≥ 3 additional risk factors; 6, assessment of outcome (independent blind assessment or record linkage); 7, follow-up ≥ 62 m; 8, complete accounting for cohorts or subjects lost to follow-up unlikely to introduce bias.

“☆”was scored 1 and “-” was scored “0”.

### Primary outcomes

**Fig. 2:**
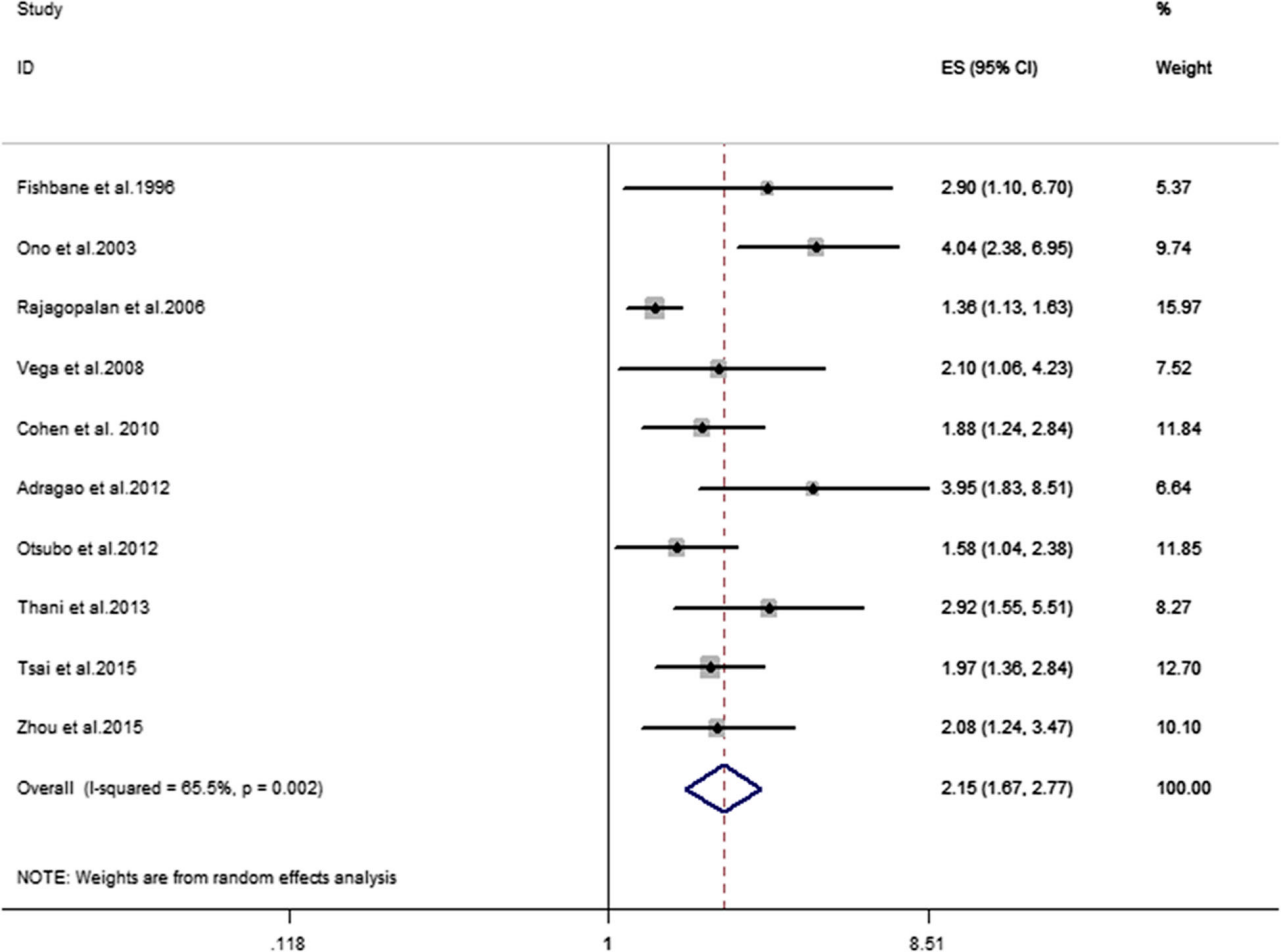
Association between PAD and risk of all-cause mortality in HD patients

**Fig. 3:**
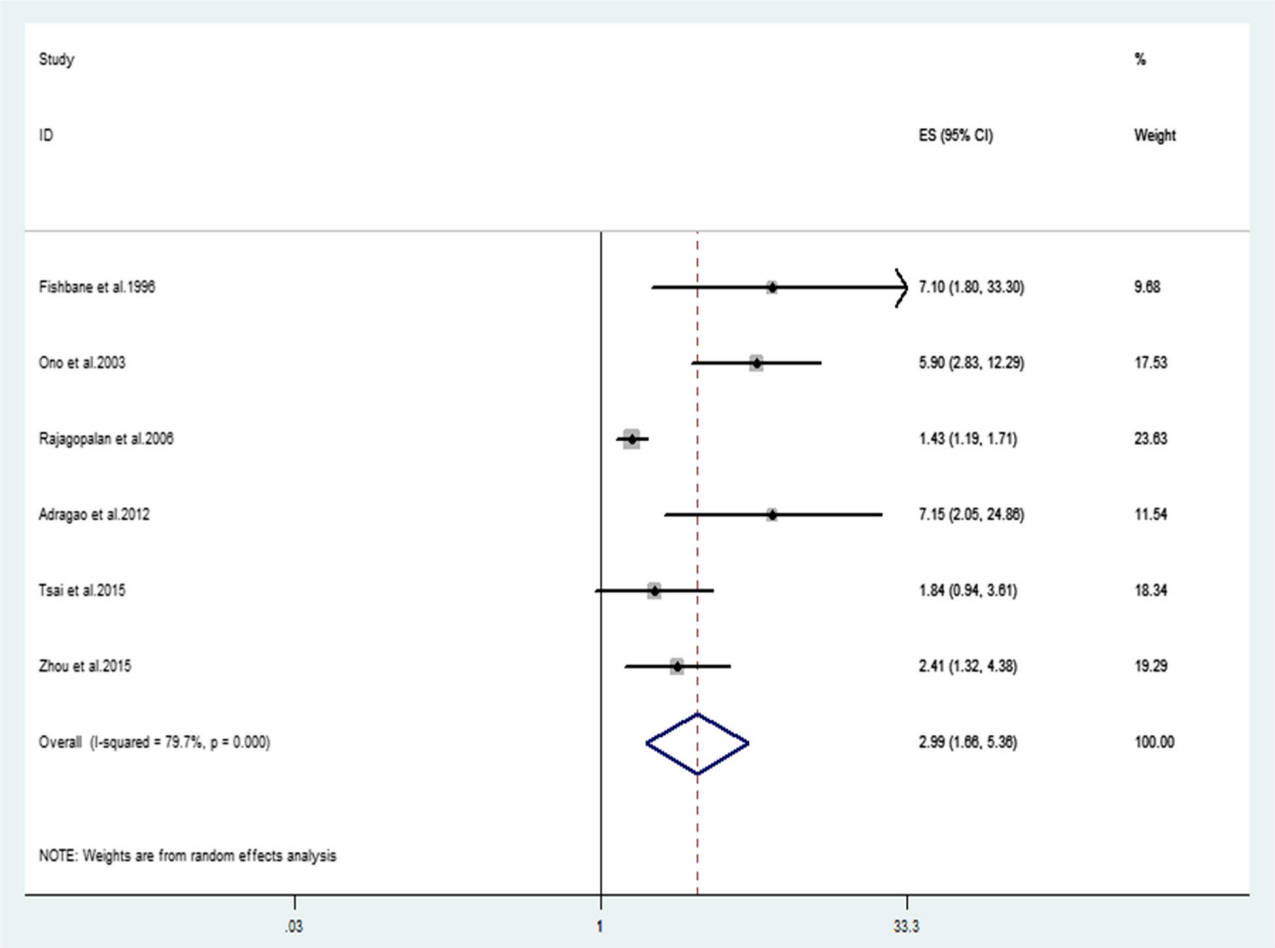
Association between PAD and risk of CV mortality in HD patients

The analysis pooled data for all-cause mortality from 10 studies (weights: 5.37%–15.97%) and CV mortality from 6 studies (weights, 9.68%–23.63%). Both data sets were heterogeneous (all-cause mortality, I^2^ = 65.5% and CV mortality, I^2^ = 79.7%), therefore, the analyses used random effects models. Overall, PAD was associated with an increased all-cause mortality after multivariate adjustment (RR: 2.15, 95% CI 1.67–2.77) (Fig. 2). Meanwhile, PAD significantly increased CV mortality (RR: 2.99, 95%CI 1.65-5.36) (Fig. 3). The associations remained significant after omitting any single study conforming to Jackknife sensitivity analysis (All-cause mortality: Additional file 3 and CV mortality: Additional file 4). There was publication bias identified by Egger’s test for all-cause mortality (*P* = 0.002) (Additional file 5) and CV mortality (*P* = 0.015) (Additional file 6).

### Subgroup meta-analysis

For all-cause mortality, we further conducted subgroup meta-analysis. Table 3 showed possible confounding factors and outcomes. In the subgroups of prospective study, retrospective study, follow-up time < 62 months, follow-up time ≥ 62 months, prevalence of diabetes mellitus (%) < 29, prevalence of PAD (%) ≥ 26, duration of dialysis < 9.4 years, and duration of dialysis ≥ 9.4 years, no statistical heterogeneity was detected (all *P* values > 0.1). The observed positive association was more pronounced among prospective studies compared to retrospective studies, and more pronounced among studies with shorter follow-up time than studies with longer follow-up time. Additionally, the association between PAD and all-cause mortality significantly differed according to the study design (*P* = 0.029 for the interaction) and follow-up time (*P* = 0.029 for the interaction).

**Table 3:** Subgroup meta-analysis for all-cause mortality

Subgroup	No. of studies	Pooled RR (95 % CI)	I^2^ (%)	*P* ^a^	*P* ^b^
Study design					
Prospective	6	2.67 (2.01, 3.55)	28.7	0.220	0.029
Retrospective	4	1.58 (1.28, 1.93)	28.1	0.243	
Ascertainment of PAD with ABI only					
Yes	4	2.33 (1.53, 3.53)	62.9	0.044	0.649
No	6	2.05 (1.48, 2.83)	64.1	0.016	
Follow-up time, months					
<62	6	2.72 (2.04, 3.64)	52.6	0.121	0.029
≥62	4	1.61 (1.30, 2.00)	37.1	0.189	
Diabetes mullitus(%)					
<29	5	2.13(1.58, 2.87)	18.2	0.299	0.940
≥29	4	2.25(1.38, 3.68)	84.2	0.000	
PAD (%)					
<26	6	1.92 (1.45, 2.55)	70.7	0.004	0.152
≥26	4	2.85 (1.98,4.10)	0	0.694	
Duration of dialysis, years					
<9.4	6	2.58 (1.97, 3.37)	28.9	0.218	0.124
≥9.4	2	1.72 (1.29, 2.31)	0	0.561	

^a^ *P* value for heterogeneity within each subgroup. ^b^ *P* value for heterogeneity between subgroups in the meta-regression analysis

For CV mortality, we also conducted the subgroup meta-analysis (Table 4). The possible confounding factors were the same as that of all-cause mortality. All these subgroups without statistical heterogeneity in all-cause mortality were detected without statistical heterogeneity (all *P* values > 0.1) either. Silmilarly, the observed positive association was more pronounced among prospective studies compared to retrospective studies, and more pronounced among studies with shorter follow-up time than studies with longer follow-up time. And the association between PAD and CV mortality significantly differed according to the study design (*P* = 0.048 for the interaction) and follow-up time (*P* = 0.017 for the interaction). No heterogeneity with each group stratified by duration of dialysis was observed (both I^2^ = 0; *P* = 0.759 and 0.559, respectively), however, the difference between the two pooled RRs was not significant (*P* = 0.115).

**Table 4:** Subgroup meta-analysis for CV mortality

Subgroup	No. of studies	RR (95 % CI)	I^2^ (%)	*P* ^*a*^	*P* ^*b*^
Study design					
Prospective	4	4.45 (2.43, 8.16)	42.9	0.154	0.048
Retrospective	2	1.45 (1.22, 1.73)	0	0.478	
Ascertainment of PAD with ABI only					
Yes	3	3.87 (1.55, 9.63)	68.5	0.042	0.493
No	3	2.33 (1.15, 4.72)	76.7	0.014	
Follow-up time, months					
<62	3	6.33 (3.54, 11.32)	0	0.953	0.017
≥62	3	1.65 (1.22, 2.24)	33.4	0.223	
Diabetes mullitus(%)					
<29	3	4.07 (1.81, 9.16)	44.4	0.166	0.389
≥29	3	2.36 (1.07, 5.22)	85.4	0.001	
PAD (%)					
<26	4	2.33 (1.30, 4.17)	80.7	0.001	0.160
≥26	2	7.13 (2.76, 18.4)	0	0.994	
Duration of dialysis, years					
<6.9	2	6.20 (3.29, 11.67)	0	0.795	0.115
≥6.9	2	2.14(1.37, 3.34)	0	0.559	

^a^ *P* value for heterogeneity within each subgroup. ^b^ *P* value for heterogeneity between subgroups in the meta-regression analysis

## Discussion

To our knowledge, this current study is the first meta-analysis to synthesize the association between PAD and risk of all-cause and CV mortality in HD patients. We confirmed that PAD was independently associated with a 115% higher adjusted RR for all-cause mortality on a total of available 10 studies with 32,864 HD patients. Moreover, for the CV mortality, a 199% higher risk was yield on a total of 6 studies with 31,794 HD patients. There was heterogeneity among the studies included. Study design and follow up time were found to be two potential sources of heterogeneity for all-cause and CV mortality.

The prevalence of PAD was higher in HD patients than in general population. In our included 10 studies, the highest prevalence of PAD was 41% and average prevalence was 26.0%. The prevalence of PAD was 3% ~ 10% in the general population [22]. This may be explained by several reasons: Firstly, it may be related to the old age in HD patients. PAD mainly occurred after the age of 50 years old. The prevalence rose with age [23]. It was 15% ~ 20% in persons > 70 years old [22] and by the age of 80, the prevalence was around 20% [23]. Of note, the mean ages in our 10 studies ranged from 56.4 to 65 years old. So the prevalence in our study may be higher than it in general population. Secondly, in patients on maintenance HD, the immune disorders and chronic inflammation were prevalent [24, 25]. They were mediated by inflammatory factors, such as β_2_-Microglobulin, cystatin C and hsCRP and so on [26–32]. These factors acted on blood vessels persistently and collectively to disorder the function of vascular endothelial cell and to lead to atherogenesis eventually. And oxidative stress injury and hypercoagulable states also played a role in development of PAD [7]. Thirdly, some uremic toxins, such as asymmetric dimethylarginine, could not be cleared up by HD. These cumulative toxins would have a bad effect on vascular endothelial cell and lead to atherogenesis [33]. Finally, renal failure can result in reduction of active vitamin D in blood. The shortage of vitamin D played a part in the progress of subclinical arteriosclerosis [34]. Of note, the mechanism of PAD in HD patients is complex and need to be explored in further pathophysiologic studies.

Our study demonstrated that PAD was associated with increased mortality risk, which may be due to the following reasons. Firstly, PAD is the narrow and obstructive lesions happened to aorta and limb blood supply arteries usually secondary to atherosclerosis [7]. Ischemia and necrosis were prevalent as a result of blockage of blood supply to organs (except for the heart), limbs and torso. Secondly, PAD was associated with other CV risk factors, such as diabetes, dyslipidemia, hypertension, and smoking [23, 35]. Thirdly, PAD led to a limitation in exercise performance, which may cause the progression of atherosclerosis [36].

Fortunately, several treatments have shown beneficial effects on the survival of patients with PAD. Firstly, antiplatelet treatment had a 23% reduction of serious vascular events for PAD patients, which was indicated by a meta analysis of 42 randomized clinical trials including 9214 patients with PAD [37]. Secondly, the use of statin drugs could reduce CV diseases fatality rate and all-cause mortality [38, 39]. Thirdly, quitting smoking lead to a lower 5-year mortality in patients with PAD [40]. However, there were no relevant clinical trials in HD patients. There was only a pilot trial showed that prostaglandin I_2_ analog might improve symptoms of PAD in HD patients, but did not report relevant mortality risk [41]. We are looking forward to high-quality randomized clinical trials in this area.

Diabetes mellitus may have an effect on the mortality risk of PAD in HD patients. 7 included articles showed that prevalence of diabetes mellitus was significantly higher in HD patients with PAD than without PAD [9, 10, 13–17]. However, in our analysis, no difference of the observed positive association was showed between the high and low prevalence of diabetes mellitus in the HD populations. Thani et al. revealed that PAD had a 2.45-fold increased mortality in HD patients with diabetes and a 0.67-fold increased mortality in HD patients without diabetes [42]. More similarly designed studies are needed to explore the effect of diabetes mellitus in this topic.

PAD is diagnosed by an ABI value < 0.9 in either leg. Besides, typical manifestations including intermittent claudication and critical limb ischemia, and existence of vascular obstruction based on ultrasonography or angiography can also be used to diagnose PAD. ABI is calculated by the ankle systolic pressure divided by the arm systolic pressure. The two systolic pressures are measured simultaneously when the patients have a rest in a supine position for 5 min. ABI measurement is simple, noninvasive and cheap to be performed. Given the simple diagnosis and the potential risk for mortality, PAD can be used as a convenient monitoring point for survival of HD patients. Especially for those undergoing HD with hypertension, diabetes, dyslipidemia, smoking, and age > 50 years, screening for and management of PAD may achieve higher cost-effectiveness.

The main strength of our study were all-inclusive available studies about theme of interest and the sufficient consideration of potentially relevant confounding factors in every article included. However, there were several potential limitations as below. Firstly, subgroup analysis found that for prospective studies (*n* = 6) there was a 167% increase all-cause mortality, but for retrospective studies (*n* = 4) only 58% increase was shown. The difference was obvious and statistically significant. Potentially, data for retrospective studies were retrieved from existing records which may not contain detailed clinical information. So as to assess the association more precisely, we look forward to more mass prospective studies. Secondly, existence of potential publication bias may lead to our exaggerating estimation of the association between PAD and mortality risk. Finally, the diagnosis criteria for PAD at baseline were not completely uniform. For example, Rajagopalan et al. [10] diagnosed PAD just by history and clinical assessment and left out ABI measurement, which may result in lower positive ratio of PAD patients compared with the other studies using ABI. Meanwhile, ABI is now widely used to diagnose PAD, which may result in the early diagnosis of PAD, therefore it might have some bias in our final conclusion.

## Conclusion

Our meta-analysis suggests PAD is associated with increased risks of both all-cause and CV mortality in patients on HD. For HD patients, this study may declare the importance of PAD and bring out a risk factor for poor prognosis. Additionally, the methods for PAD screening are inexpensive, simple and noninvasive. PAD deserves to be paid more attentions in HD patients. Since there was a moderate to significant heterogeneity among studies and confounding factors, more well-designed prospective studies and randomized clinical trials are needed in this area.

## Additional files

Additional file 1: PRISMA checklist. (DOC 53 kb)
Additional file 2: Search strategy. (DOCX 13 kb)
Additional file 3: Sensitivity analysis for all-cause mortality. (TIF 6154 kb)
Additional file 4: Sensitivity analysis for CV mortality. (TIF 9857 kb)
Additional file 5: Egger’s test for all-cause mortality. (JPG 99 kb)
Additional file 6: Egger’s test for CV mortality. (PNG 197 kb)

## Abbreviations

ABI: Ankle-brachial blood pressure index
AC: All-cause
CI: 95% confidence intervals
CV: Cardiovascular
ESRD: End-stage renal disease
HD: Hemodialysis
HR: Hazard ratio
NOS: Newcastle-ottawa scale
PAD: Peripheral arterial disease
PRISMA: Preferred reporting items for systematic reviews and meta-analyses
RR: Risk ratio

## References

[1] Robinson BM, Akizawa T, Jager KJ, Kerr PG, Saran R, Pisoni RL (2016). Factors affecting outcomes in patients reaching end-stage kidney disease worldwide: differences in access to renal replacement therapy, modality use, and haemodialysis practices. Lancet.

[2] Masakane I, Nakai S, Ogata S, Kimata N, Hanafusa N, Hamano T, Wakai K, Wada A, Nitta K (2015). An overview of regular dialysis treatment in Japan (as of 31 December 2013). Ther. Apher. Dial..

[3] Yan G, Norris KC, Xin W, Ma JZ, Yu AJ, Greene T, Yu W, Cheung AK (2013). Facility size, race and ethnicity, and mortality for in-center hemodialysis. J Am Soc Nephrol.

[4] Bellasi A, Kooienga L, Block GA, Veledar E, Spiegel DM, Raggi P (2009). How long is the warranty period for nil or low coronary artery calcium in patients new to hemodialysis?. J Nephrol.

[5] Stenvinkel P, Heimburger O, Paultre F, Diczfalusy U, Wang T, Berglund L, Jogestrand T (1999). Strong association between malnutrition, inflammation, and atherosclerosis in chronic renal failure. Kidney Int.

[6] Patel MR, Conte MS, Cutlip DE, Dib N, Geraghty P, Gray W, Hiatt WR, Ho M, Ikeda K, Ikeno F (2015). Evaluation and treatment of patients with lower extremity peripheral artery disease: consensus definitions from Peripheral Academic Research Consortium (PARC). J Am Coll Cardiol.

[7] Krishna SM, Moxon JV, Golledge J (2015). A review of the pathophysiology and potential biomarkers for peripheral artery disease. Int J Mol Sci.

[8] Fishbane S, Youn S, Flaster E, Adam G, Maesaka JK (1996). Ankle-arm blood pressure index as a predictor of mortality in hemodialysis patients. Am J Kidney Dis.

[9] Ono K (2003). Ankle-brachial blood pressure index predicts All-cause and cardiovascular mortality in hemodialysis patients. J Am Soc Nephrol.

[10] Rajagopalan S, Dellegrottaglie S, Furniss AL, Gillespie BW, Satayathum S, Lameire N, Saito A, Akiba T, Jadoul M, Ginsberg N (2006). Peripheral arterial disease in patients with end-stage renal disease: observations from the Dialysis Outcomes and Practice Patterns Study (DOPPS). Circulation.

[11] Cohen LM, Ruthazer R, Moss AH, Germain MJ (2010). Predicting six-month mortality for patients who are on maintenance hemodialysis. Clin J Am Soc Nephrol.

[12] Adragao T, Pires A, Branco P, Castro R, Oliveira A, Nogueira C, Bordalo J, Curto JD, Prata MM (2012). Ankle--brachial index, vascular calcifications and mortality in dialysis patients. Nephrol Dial Transplant.

[13] Otsubo S, Kitamura M, Wakaume T, Yajima A, Ishihara M, Takasaki M, Ueda S, Sugimoto H, Otsubo K, Kimata N (2012). Association of peripheral artery disease and long-term mortality in hemodialysis patients. Int Urol Nephrol.

[14] Al Thani H, El-Menyar A, Hussein A, Sadek A, Sharaf A, Singh R, Koshy V, Al Suwaidi J (2013). Prevalence, predictors, and impact of peripheral arterial disease in hemodialysis patients: a cohort study with a 3-year follow-up. Angiology.

[15] Tsai MH, Liou HH, Leu JG, Yen MF, Chen HH (2015). Sites of peripheral artery occlusive disease as a predictor for all-cause and cardiovascular mortality in chronic hemodialysis. PLoS One.

[16] Zhou Y, Zhang J, Zhu M, Lu R, Wang Y, Ni Z (2015). Plasma pentraxin 3 is closely associated with peripheral arterial disease in hemodialysis patients and predicts clinical outcome: a 6-year follow-Up. Blood Purif.

[17] Vega A, Pérez García R, Abad S, Verde E, López Gómez JM, Jofré R, Puerta M, Rodríguez P (2008). Enfermedad vascular periférica: prevalencia, mortalidad y asociación con inflamación en hemodiálisis. Nefrología.

[18] Moher D, Liberati A, Tetzlaff J, Altman DG, Group P (2009). Preferred reporting items for systematic reviews and meta-analyses: the PRISMA statement. Ann Intern Med.

[19] Walls GA SB, O'Connell D, Peterson J, et al. The Newcastle-Ottawa Scale (NOS) for assessing the quality of nonrandomised studies in meta-analyses. Available online: http://www.ohri.ca/programs/clinical_epidemiology/oxford.asp.

[20] Higgins JP, Thompson SG, Deeks JJ, Altman DG (2003). Measuring inconsistency in meta-analyses. BMJ.

[21] DerSimonian R, Laird N (1986). Meta-analysis in clinical trials. Control Clin Trials.

[22] Dua A, Lee CJ (2016). Epidemiology of Peripheral Arterial Disease and Critical Limb Ischemia. Tech Vasc Interv Radiol.

[23] Criqui MH, Aboyans V (2015). Epidemiology of peripheral artery disease. Circ Res.

[24] Sharif MR, Chitsazian Z, Moosavian M, Raygan F, Nikoueinejad H, Sharif AR, Einollahi B (2015). Immune disorders in hemodialysis patients. Iran. J. Kidney Dis..

[25] Stenvinkel P (2001). Inflammatory and atherosclerotic interactions in the depleted uremic patient. Blood Purif.

[26] Ozaki Y, Imanishi T, Akasaka T (2015). Inflammatory biomarkers in peripheral artery disease: diagnosis, prognosis, and therapeutic challenges. Curr Med Chem.

[27] Stone PA, Yacoub M (2014). Inflammatory biomarkers in peripheral arterial disease. Semin Vasc Surg.

[28] Fung ET, Wilson AM, Zhang F, Harris N, Edwards KA, Olin JW, Cooke JP (2008). A biomarker panel for peripheral arterial disease. Vasc Med.

[29] Hiatt WR, Zakharyan A, Fung ET, Crutcher G, Smith A, Stanford C, Cooke J (2012). A validated biomarker panel to identify peripheral artery disease. Vasc Med.

[30] Kals J, Zagura M, Serg M, Kampus P, Zilmer K, Unt E, Lieberg J, Eha J, Peetsalu A, Zilmer M (2011). beta2-microglobulin, a novel biomarker of peripheral arterial disease, independently predicts aortic stiffness in these patients. Scand J Clin Lab Invest.

[31] Joosten MM, Pai JK, Bertoia ML, Gansevoort RT, Bakker SJ, Cooke JP, Rimm EB, Mukamal KJ. beta2-microglobulin, cystatin C, and creatinine and risk of symptomatic peripheral artery disease. Available online: http://jaha.ahajournals.org/content/3/4/e000803. 10.1161/JAHA.114.000803 PMC4310365 24980133

[32] Ridker PM, Stampfer MJ, Rifai N (2001). Novel risk factors for systemic atherosclerosis: a comparison of C-reactive protein, fibrinogen, homocysteine, lipoprotein(a), and standard cholesterol screening as predictors of peripheral arterial disease. Jama.

[33] Kielstein JT, Zoccali C (2005). Asymmetric dimethylarginine: a cardiovascular risk factor and a uremic toxin coming of age?. Am J Kidney Dis.

[34] Reis JP, von Mühlen D, Michos ED, Miller ER, Appel LJ, Araneta MR, Barrett-Connor E (2009). Serum vitamin D, parathyroid hormone levels, and carotid atherosclerosis. Atherosclerosis.

[35] Cheung AK, Sarnak MJ, Yan G, Dwyer JT, Heyka RJ, Rocco MV, Teehan BP, Levey AS (2000). Atherosclerotic cardiovascular disease risks in chronic hemodialysis patients. Kidney Int.

[36] Olin JW, White CJ, Armstrong EJ, Kadian-Dodov D, Hiatt WR (2016). Peripheral artery disease: evolving role of exercise, medical therapy, and endovascular options. J Am Coll Cardiol.

[37] Antithrombotic Trialists C (2002). Collaborative meta-analysis of randomised trials of antiplatelet therapy for prevention of death, myocardial infarction, and stroke in high risk patients. BMJ.

[38] Group HPSC. MRC/BHF Heart Protection Study of cholesterol lowering with simvastatin in 20 536 high-risk individuals: a randomised placebocontrolled trial. The Lancet. 2002;360(9326):7–22. 10.1016/S0140-6736(02)09327-3 12114036

[39] Heart Protection Study Collaborative G (2007). Randomized trial of the effects of cholesterol-lowering with simvastatin on peripheral vascular and other major vascular outcomes in 20,536 people with peripheral arterial disease and other high-risk conditions. J Vasc Surg.

[40] Armstrong EJ, Wu J, Singh GD, Dawson DL, Pevec WC, Amsterdam EA, Laird JR (2014). Smoking cessation is associated with decreased mortality and improved amputation-free survival among patients with symptomatic peripheral artery disease. J Vasc Surg.

[41] Ohtake T, Sato M, Nakazawa R, Kondoh M, Miyaji T, Moriya H, Hidaka S, Kobayashi S (2014). Randomized pilot trial between prostaglandin I2 analog and anti-platelet drugs on peripheral arterial disease in hemodialysis patients. Ther Apher Dial.

[42] Al-Thani H, Shabana A, Hussein A, Sadek A, Sharaf A, Koshy V, El-Menyar A (2015). Cardiovascular complications in diabetic patients undergoing regular hemodialysis: a 5-year observational study. Angiology.

