# Supplementary material for: Association of peripheral arterial disease with all-cause and cardiovascular mortality in hemodialysis patients: a meta-analysis
Source: BMC Nephrol. 2016 Nov 25;17:195. doi: 10.1186/s12882-016-0397-1 (PMC5124247; doi:10.1186/s12882-016-0397-1)
Supplement: Additional file 2: — Search strategy. (DOCX 13 kb) [file 12882_2016_397_MOESM2_ESM.docx]

Pubmed:

Search **(((((((Mortality) OR Death) OR Outcome) OR Prognos*)) AND (((peritoneal dialysis) OR hemodialysis) OR dialysis)) AND (((((((((((Peripheral artery disease) OR Peripheral arterial disease) OR PAD) OR Claudication) OR limb ischaemia) OR limb ischemia) OR ABI) OR ABPI) OR ankle–brachial index) OR Ankle-brachial blood pressure index) OR peripheral arterial occlusive disease))**Filters: **Humans**

**Web of science^TM^**

[TS=((((((Mortality) OR (Death)) OR (Outcome)) OR (Prognos*)) AND ((((peritoneal) (dialysis)) OR (hemodialysis)) OR (dialysis))) AND ((((((((((((Peripheral) (artery) (disease)) OR ((Peripheral) (arterial) (disease))) OR (PAD)) OR (Claudication)) OR ((limb) (ischaemia))) OR ((limb) (ischemia))) OR (ABI)) OR (abpa)) OR ((ankle) (brachial) (index))) OR ((Ankle-brachial) (blood) (pressure) (index))) OR ((peripheral) (arterial) (occlusive) (disease))))](http://apps.webofknowledge.com/UseSpellSuggestion.do?action=takeSuggestion&product=WOS&SID=4AZRm2SqYYaBq5rM7gt&search_mode=AdvancedSearch&update_back2search_link_param=yes&viewType=summary&qid=6)

EMBASE:

1 Peripheral artery disease

2 Peripheral arterial disease

3 PAD

4 Claudication

5 limb ischaemia

6 limb ischemia

7 ABI

8 ABPI

9 ankle–brachial index

10 Ankle-brachial blood pressure index 
11 peripheral arterial occlusive disease 
12 1 or 2 or 3 or4 or 5 or 6 or 7 or 8 or 9 or 10 or 11
13peritoneal dialysis
14 hemodialysis
15 dialysis

16 13 or 14 or 15

17 Mortality

18 Death

19 Outcome

20 Prognosis

21 17 or 18 or 19 or 20

22 12 and 16 and 21

Cochrance Library

#1 Peripheral artery disease or Peripheral arterial disease or PAD  or Claudication or limb ischaemia (Word variations have been searched)

#2 limb ischemia  or ABI or ABPI or ankle–brachial index or Ankle-brachial blood pressure index  (Word variations have been searched)

#3 peripheral arterial occlusive disease  (Word variations have been searched)

#4 peritoneal dialysis or hemodialysis or dialysis (Word variations have been searched)

#5 Mortality or Death or Outcome or Prognos*  (Word variations have been searched)

#6 #1 or #2 or #3

#7 #4 and #5 and #6

Choose “trials”
