# Supplementary figures and images for: Association of peripheral arterial disease with all-cause and cardiovascular mortality in hemodialysis patients: a meta-analysis
Source: BMC Nephrol. 2016 Nov 25;17:195. doi: 10.1186/s12882-016-0397-1 (PMC5124247; doi:10.1186/s12882-016-0397-1)

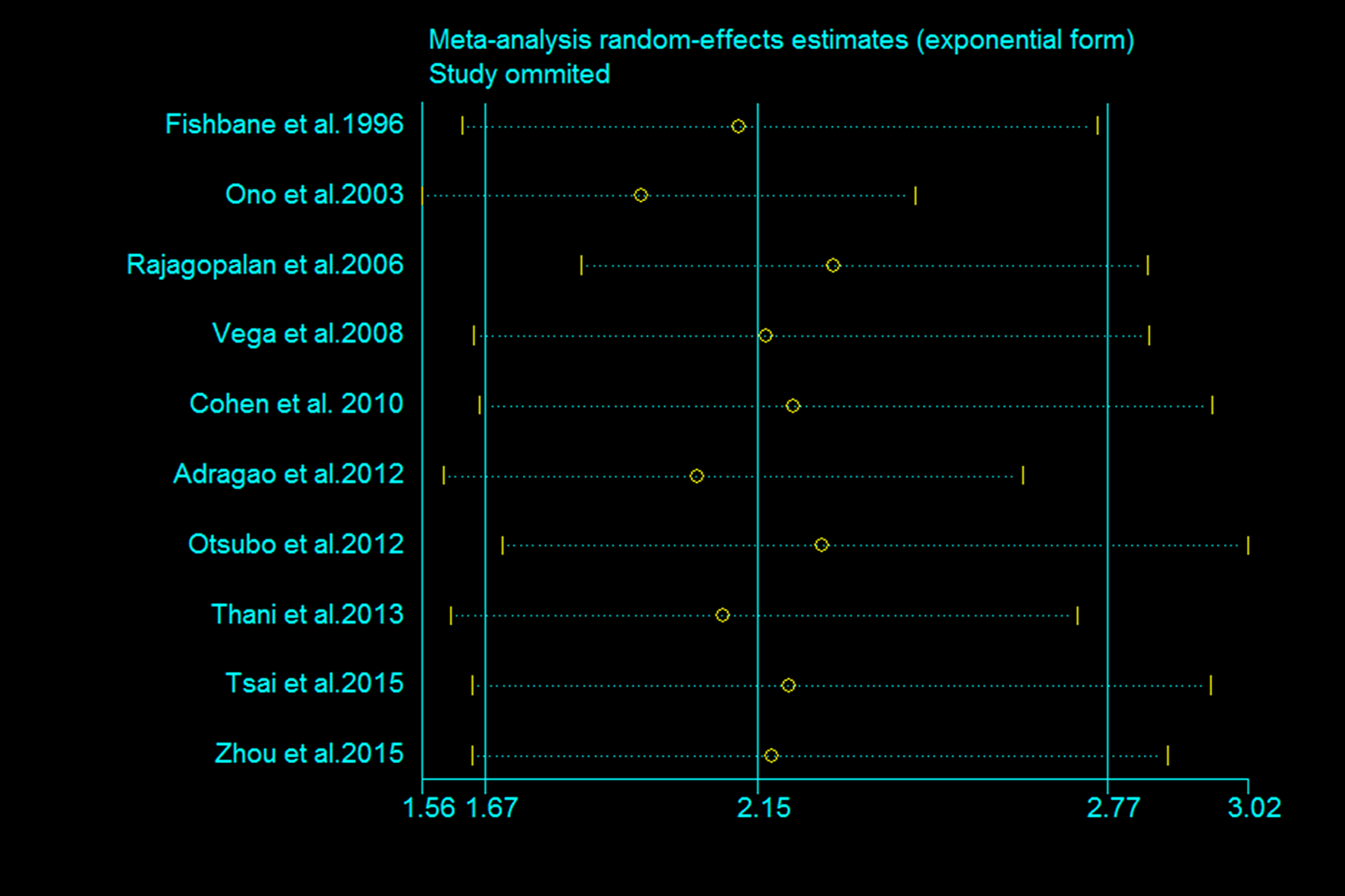

Supplement: Additional file 3: — Sensitivity analysis for all-cause mortality. (TIF 6154 kb) [file 12882_2016_397_MOESM3_ESM.tif]

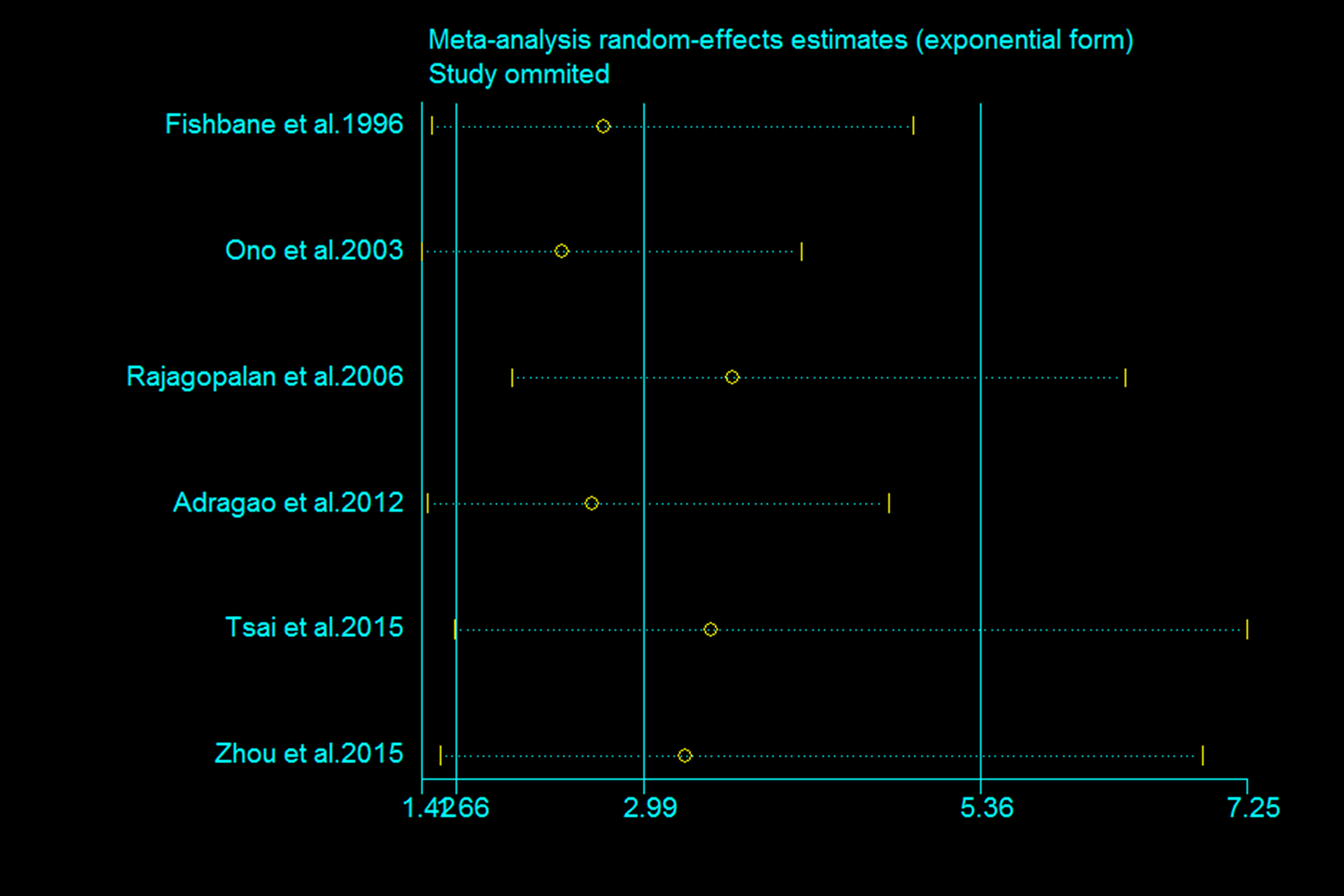

Supplement: Additional file 4: — Sensitivity analysis for CV mortality. (TIF 9857 kb) [file 12882_2016_397_MOESM4_ESM.tif]

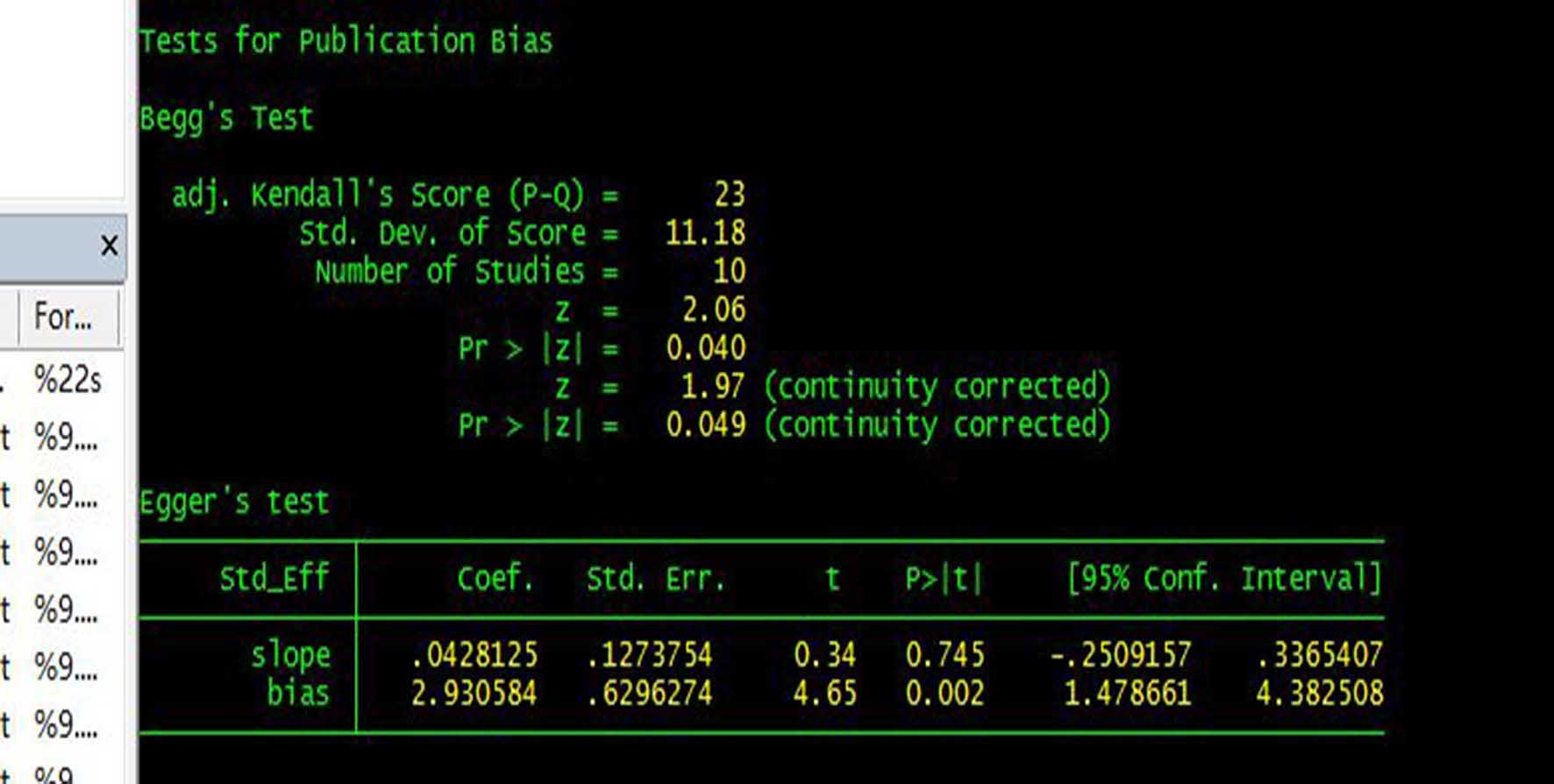

Supplement: Additional file 5: — Egger’s test for all-cause mortality. (JPG 99 kb) [file 12882_2016_397_MOESM5_ESM.jpg]

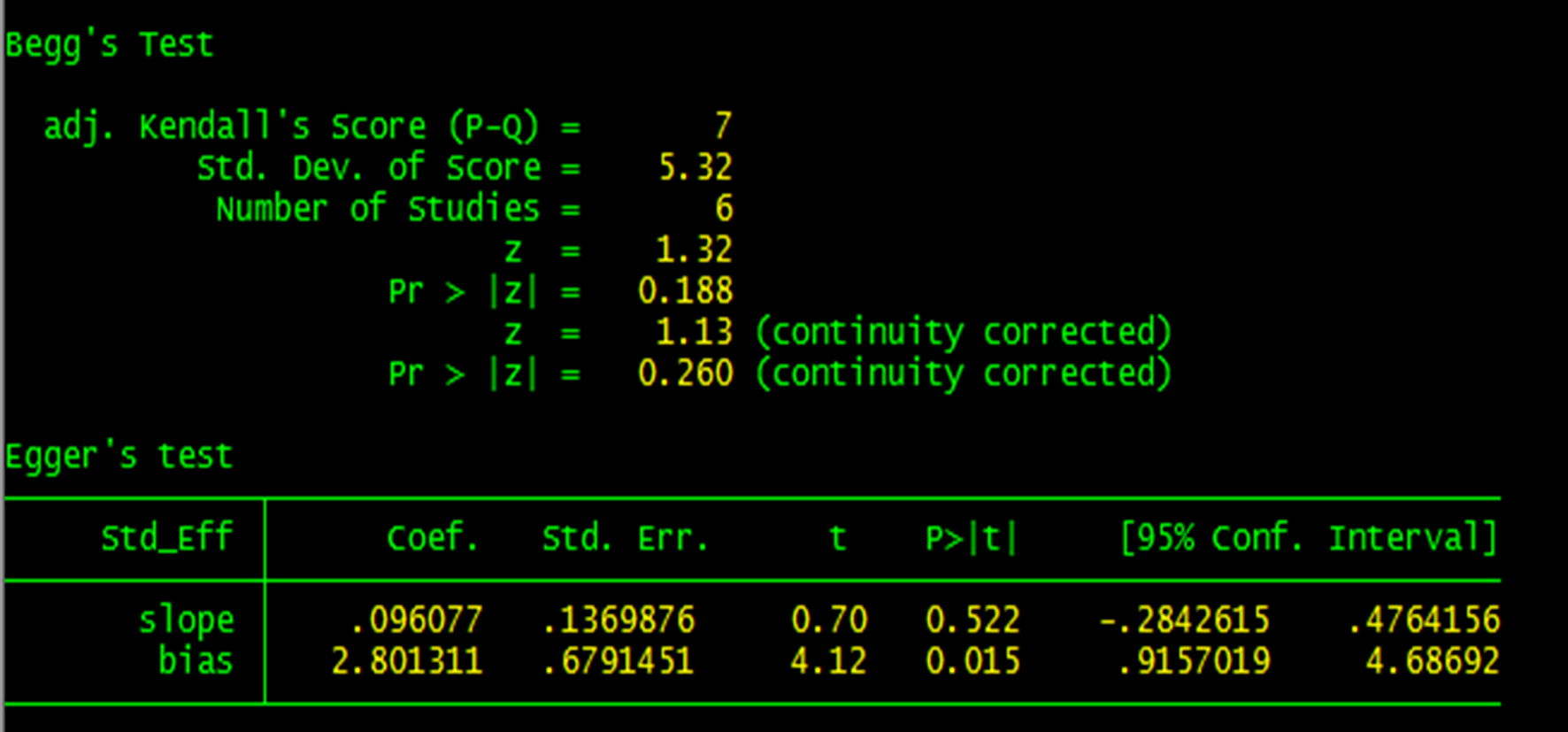

Supplement: Additional file 6: — Egger’s test for CV mortality. (PNG 197 kb) [file 12882_2016_397_MOESM6_ESM.png]
